# Effectiveness and implementation of interventions to increase commuter cycling to school: a quasi-experimental study

**DOI:** 10.1186/s12889-015-2536-1

**Published:** 2015-11-30

**Authors:** Lars Østergaard, Jan Toftegaard Støckel, Lars Bo Andersen

**Affiliations:** Center for Research in Childhood Health, Department of Sports Science and Clinical Biomechanics, University of Southern Denmark, Campusvej, 555230 Odense, Denmark

**Keywords:** Fitness, Adiposity, Bicycling, Children, Injury

## Abstract

**Background:**

Active transportation to school has been positively associated with various health parameters whereas only sparse evidence exists on risk of injury while commuting to school. This study investigated the overall effectiveness of cycling promotion combined with structural changes on cycling to school.

**Methods:**

Interventions at public schools in three different regions in Denmark were based on planned infrastructural changes near schools (e.g. road surface and traffic regulation) and school-motivation for promoting commuter cycling. Participants were pupils from control schools (*n* = 12) or intervention schools (*n* = 13). All children (*n* = 2415) from the 4^th^ and 5^th^ grade were measured at baseline during spring 2010 and at follow-up one year later.

**Results:**

No significant differences in commuter cycling were detected in the adjusted analyses comparing the intervention with the control group neither when assessed as changes in short term (beta: 0.15 trips/week, *p* = 0.463) nor when assessed as changes in long term school cycling (beta: −0.02 units, *p* = 0.485). No differences were observed neither in the incidence of traffic injuries nor in the characteristics of injuries when comparing the control group and the intervention group. Approximately 50 % of all traffic injuries occurred during school transport with most injuries categorized as solo injuries. The only significant predictor of future traffic injuries was previous school transport injuries.

**Conclusion:**

This multifaceted school cycling promotion programme did not affect school cycling behaviour or the health parameters assessed. Implementation issues relevant in the planning of future school cycling interventions are discussed in the article. The one year incidence of being involved in a traffic injury was approximately 25 % with almost 50 % of all traffic injuries occurred during school transport. Previous school transport injury predicted future school traffic injuries.

**Electronic supplementary material:**

The online version of this article (doi:10.1186/s12889-015-2536-1) contains supplementary material, which is available to authorized users.

## Background

A bulk of evidence indicates that active transportation to school such as walking and cycling may be a substantial and important part of the total amount of children’s daily physical activity. A multitude of observational studies have found that cycling as active transportation to school is positively associated with e.g. weight status [1, 2], muscular fitness [3, 4] and cardiorespiratory fitness [4–6]. Supportive findings have been reported in an efficacious intervention study concluding that cycling to school counteracted a clustering of cardiometabolic risk factors [7].

Very different approaches including infrastructural changes, marketing programs and policy changes have been made in the general attempt of increasing cycling [8] and a range of different studies have investigated the effectiveness of different interventions aimed at increasing commuter cycling [9, 10].

A range of studies have investigated the effectiveness of interventions to promote physical activity in children [11] but it is obvious that experimental active school transportation studies though requested almost a decade ago [12] are lacking. Also it is obvious that all previous published active school transportation effectiveness studies have evaluated interventions aimed at both walking and cycling to school [13, 14].

Keeping in mind that walking and cycling to school are two different behaviours with specific determinants [15], we constructed a multifaceted intervention inspired by correlates of cycling to school [16, 17] and focused e.g. on cycling skills and physical environmental characteristics. A package of interventions was offered since it has been suggested that increases in cycling require different complimentary interventions [8].

The main aim of this study was to assess the effectiveness of the school cycling promotion programme entitled “Tryg og Sikker Skolecykling” (Safe and secure cycling to school) on school cycling while also assessing potential concomitant health effects. Furthermore, since cycling-related injuries are often thought to supersede the preventive health beneficial effects [18] the secondary aim of this study was to quantify the incidence, the predictors and the number of injuries related to cycling to school.

## Methods

### Study design

Public schools in the municipality of Copenhagen, the municipality of Fredericia and schools on the island of Funen were, based on the existence of local plans for infrastructural changes near schools and school motivation for implementing school cycling interventions, included into this quasi-experimental study as either control schools (*n* = 12) or intervention schools (*n* = 13) by the Danish Cyclists Federation. It was required that the control schools were not involved in any physical activity promotion projects during the study period. School recruitment was completed prior to the start of baseline data collection in April and May 2010 and follow-up measurements were conducted one year later. At baseline all children (*n* = 2415) from the 4^th^ and 5^th^ grade were included into the study using public school registrations. Though the school registrations were obtained just ahead of conducting the baseline measurements, a few children (*n* = 14) had entered or left the schools leaving a total sample of 2,401 children at baseline. The study was granted an exemption from requiring ethics approval of the Regional Scientific Ethical Committees for Southern Denmark and no parental consent was required. We obtained passive parental consent by distributing a letter to all parents with information about the study including the measures conducted. All parents were encouraged to contact the project leader in case they needed any clarification. This approach for parental consent was appropriate since the project and project evaluation (e.g. fitness measures) was a part of the mandatory school curriculum. Additionally it should be mentioned that the study was approved by the Danish Data Protection Agency and rules regarding data security and anonymization followed.

The cycling incentives at the intervention schools consisted of “hard” interventions planned ahead of this study by the local authorities and “soft” interventions which were initiatives implemented by The Danish Cyclists Federation (for details see Additional file 1: Table B). “Hard” interventions included structural changes near the school in e.g. road surface, signposting and traffic regulation such as one-way streets and regulation of car drop off zones whereas “soft” interventions generally focused on increasing school cycling motivation (e.g. through competitions and monitoring) as well as cycling safety (e.g. through school traffic policy, cycle training and bicycle maintenance).

### Measurements

All tests were conducted in the school setting by test teams using a standard protocol. Questionnaires were used in order to register transportation to school, age, gender, injuries and leisure time physical activity (LTPA). All physical measurements and completion of the questionnaire were conducted at the school on the same day at both baseline and follow-up.

#### Anthropometry

Weight and height were measured in light clothing without shoes. Weight was measured to the nearest 0.1 kg with a digital Seca 813 scale (SECA GmbH, Hamburg, Germany). Height was measured to the nearest 0.5 cm with a mobile Seca 210 stadiometer (SECA GmbH, Hamburg, Germany). BMI was calculated as weight (kg) divided by the height squared (m^2^). Overweight/obesity status was defined according to age- and gender-specific published cutpoints [19].

#### Leisure time physical activity

Physical activity beyond transport to school was defined from a question on weekly hours of non-school related physical activity. The phrasing of the question was: “Which of the following best describes your level of leisure time activities?”. The following options were provided: 1) I attend sport several times per week where I train hard, 2) I attend sport about one time per week and additionally move every day, 3) I am physically active but do not attend sport in a local club, 4) I attend many forms of activity but not sport or exercise, 5) I do not move very much but often watch TV, play computer or do other sedentary activities.

#### Physical activity from cycling

Long term school cycling was assessed from the question: “How often do you cycle to or from school?” with the possible options: 1) Always or almost always, 2) Sometimes, 3) Never or hardly ever. Cycling to school last week was assessed with the question: “Think of the last week (Monday till Friday). How many days did you cycle to school?” The possible options were: 1) zero days (did not cycle), 2) one day, 3) two days, 4) three days, 5) four days, and 6) five days (cycled every day). Cycling from school last week was assessed from a virtually identical question. Cycling last week beyond school cycling was assessed with the following question: “If you neglect cycling to and from school – how much did you cycle in the last week?” 1) Often or very often, 2) Sometimes, and 3) Seldom or not at all.

#### Cardiorespiratory fitness

Aerobic capacity was measured using the Andersen test, a maximal intermittent running test which has high reproducibility (*r* = 0.84) and which in a comparable sample of children, correlate well with directly measured VO_2_max (*r* = 0.68) [20]. The test was carried out indoors as previously described by Andersen et al. 2008 and fitness calculated as: 18.38 + (0.033*distance)-(5.92*sex), boys = 0 and girls =1 [20]. At some schools (*n* = 10) a 20 m running lane was not possible and consequently the test was conducted on a 15 m lane and results adjusted (multiplied by 1.1145) according to the findings from an internal validation study where children (*n* = 41) were tested on both a 15 m and a 20 m lane.

#### Traffic injuries

One year incidence of all traffic injuries was assessed from the question: “Have you during the last year been involved in a traffic injury?” with the following options: 1) Yes, 2) No, 3) Do not know/remember. The purpose of the trip in which the incident occurred was assessed from the question: “Did the traffic injury occur on the way to school or from school?” with the following options: 1) On the way to school, 2) On the way home from school, 3) Do not know/remember, 4) In connection with something else. The transport mode of the respondent at the time of the injury was assessed from the question: “Which transport mode did you use when the injury occurred”. The transport mode of a potential counterpart was assessed from the question: “If a counterpart was involved in the injury, how was their mode of transport?”. For both questions answers were coded as: 1) walking, 2) cycling, 3) motorized,4) do not know/remember (and for the latter question also the option, “no counterpart involved”). The questions related to the transport mode of oneself and a potential counterpart was based on the coding system from the Danish emergency departments. The severity of the traffic injury was assessed from the question: “was the traffic injury so serious that you visited the emergency room” with the options: 1) no 2) yes, 3) do not know/remember.

### Statistical analyses

Differences in continuous outcomes (including ordinal variables treated as continuous) between the control group and the intervention group were tested using t-tests (Table 1) or using adjusted multiple linear regression analyses (Table 2). Chi-Square tests were used in order to test the differences in distributions in the control group compared to the intervention group (Tables 1, 3 and Additional file 1: Table A). In order to analyse changes, delta variables were derived from the difference between baseline and follow-up values. The delta variables were defined so that positive values reflect increases and vice versa. Multiple logistic regression analyses were used in case of dichotomous outcome variables in order to calculate odds ratios (Tables 2 and 4). In Table 2 the odds ratio of developing overweight was adjusted for baseline BMI, age and gender and only those subjects who were lean at baseline were included in the analyses. Chi-Square tests were used in order to test the differences in distributions in the control group compared to the intervention group (Tables 1 and 3). All analyses were conducted in 2013 using STATA IC version 11.0 with alpha =0.05.

**Table 1:** Characteristics of the control group and intervention group at baseline

	Control (*n* = 1105)	Intervention (*n* = 1296)	*P*-value	n (control / intervention)
Male gender (%)	48.8	51.1	0.261	1105/1296
Age (years)	10.9 (0.63)	11.0 (0.64)	0.023	981 / 1268
Weight (kg)	41.21 (8.83)	40.55 (9.24)	0.107	891 / 1071
Height (cm)	149.78 (7.69)	148.57 (7.97)	0.001	892 / 1087
BMI (kg∙m^−2^)	18.23 (2.84)	18.24 (2.93)	0.973	891 / 1070
Overweight (≥25 kg∙m^−2^)	17.29 %	17.37 %	0.964	781 / 1048
Obesity (≥30 kg∙m^−2^)	3.84 %	2.96 %	0.230	781 / 1048
Cardiorespiratory fitness (mL O_2_∙kg^−1^∙min^−1^)	48.07 (6.78)	49.41 (6.48)	<0.001	862 / 1065
Leisure time physical activity^a^	9.2 % 3.3 % 12.7 %	11.0 % 3.9 % 15.0 %	0.234	818 / 1117
	26.2 % 48.7 %	23.8 % 46.2 %		
Long term school cycling^b^	60.6 % 21.2 % 18.2 %	54.8 % 25.8 % 19.4 %	0.026	817 / 1117
Cycling last week beyond school cycling^c^	37.7 % 43.0 % 19.3 %	31.7 % 43.1 % 25.3 %	0.002	817 / 1117
School cycling trips last week (total number to and from school)	6.4 (4.3)	5.8 (4.4)	0.002	813 / 1113

Data are means (SD) or numbers in percent. *P*-values are for differences in distributions (Chi-squared tests) or differences in continuous outcomes (t-tests) between the control group and the intervention group

^a^From the least to the most physically active category (cf. Method section)

^b^Categories are: Always or almost always/Sometimes/Never or hardly ever”, respectively

^c^Often or very often/Sometimes/Seldom or not at all

Study conducted at three different locations in Denmark, 2010–2011

**Table 2:** Adjusted analyses of changes in leisure time physical activity, cycling behaviour, cardiorespiratory fitness and weight status

Intervention vs. control (control group as reference)	Beta-coefficient (95 % CI)	*p*-value	Number
Change in leisure time physical activity (−4;+4)	−0.09 (−0.21; 0.03)	0.124^a^	1470
Change in long term school cycling (−2;+2)	−0.02 (−0.10; 0.05)	0.485^a^	1469
Change in cycling last week beyond school cycling (−3;+3)	−0.04 (−0.14; 0.05)	0.355^a^	1469
Change in school cycling trips last week (−10;+10)	0.15 (−0.25;0.54)	0.463	1461
Change in cardiorespiratory fitness (mL O_2_∙kg^−1^∙min^−1^)	−1.45 (−1.92;-1.00)	<0.001	1335
Change in BMI (kg∙m^−2^)	0.01 (−0.13; 0.15)	0.887	1390
Odds ratio of developing overweight or obesity^b^	0.88 (0.50; 1.57)	0.675	1512

Beta-coefficients are from multiple linear regression analysis adjusted for age, gender and baseline value. All delta variables are defined so that positive values reflect increases and vice versa. ^a^The categorical variables are treated as continuous. ^b^Odds ratio is from multiple logistic regression analyses with prevalence of combined overweight and obesity at endline as the outcome adjusted for baseline BMI, age and gender (note that the control group is the reference and that this analysis included only those subjects who were lean at baseline). Study conducted at three different locations in Denmark, 2010–2011

**Table 3:** Incidence and characteristics of traffic injuries one year preceding the study and one year during the study

		Injury incidence one year prior study (assessed at baseline)			Injury incidence during study (assessed at follow-up)		
		Control (*n* = 714)	Intervention (*n* = 970)	*P*	Control (*n* = 641)	Intervention (*n* = 897)	*P*
Total injuries^a^	One year recall	166 (23.3)	231 (23.8)	0.787	151 (23.6)	216 (24.1)	0.812
	To school	39 (23.5)	54 (23.4)		35 (23.2)	33 (15.3)	
Trip destination^b^	From school	38 (22.9)	53 (22.9)	0.999	37 (24.5)	48 (22.2)	0.092
	Other/unknown	89 (53.6)	124 (53.7)		79 (52.3)	135 (62.5)	
	Walking	5 (3.0)	11 (4.7)		6 (4.0)	15 (6.9)	
	Cycling	147 (88.6)	193 (83.5)	0.465	137 (90.7)	184 (85.2)	0.251
Own transport^b^	Motorized	8 (4.8)	12 (5.2)		6 (4.0)	8 (3.7)	
	Other/unknown	6 (3.6)	15 (6.5)		2 (1.3)	9 (4.2)	
	No counterpart/solo injury	90 (54.2)	108 (46.8)		79 (52.3)	97 (44.9)	
	Walking	6 (3.6)	6 (2.6)		3 (2.0)	11 (5.1)	
Counterpart transport^b^	Cycling	41 (24.7)	65 (28.1)	0.488	42 (27.8)	62 (28.7)	0.410
	Motorized	14 (8.4)	29 (12.6)		20 (13.2)	32 (14.8)	
	Other/unknown	15 (9.0)	23 (10.0)		7 (4.6)	14 (6.5)	
Severe injuries^a^	Emergency room visits	25 (3.5)	29 (3.0)	0.556	23 (3.6)	38 (4.2)	0.521

Total and severe injuries are reported as frequency (percent relative to total number of respondents whereas all other variables are reported as frequency (percent, relative to the number of total injuries). *P*-values are from chi-squared tests of differences in distributions in the control group compared to the intervention group. ^a^ do not remember/do not know were coded as missing. ^b^ Unknown and other collapsed into one category. Study conducted at three different locations in Denmark, 2010–2011

**Table 4:** Predictors of school transport injuries - based on one year injury incidence

Class	OR	95 % CI
-5^th^ grade	Reference	
-6^th^ grade	0.96	[0.59; 1.64]
Child assessed school route safety		
-very safe	Reference	
- safe	1.14	0.75; 1.72
- unsafe or very unsafe^a^	1.02	0.46; 2.24
Parental assessed school route safety		
-very safe	Reference	
- safe	1.29	0.84; 1.96
- unsafe or very unsafe^a^	1.22	0.58; 2.52
Travel duration to school		
- 0 to 5 min	Reference	
- 6 to 15 min	1.30	0.87; 1.94
- 16 to 30 min	0.94	0.39; 2.25
- more than 30 min	1.78	0.61; 5.22
Ethnicity		
- both parents born abroad	Reference	
- one parent born in Denmark	1.46	0.72; 2.96
- both parents born in Denmark	1.32	0.76; 2.27
Previous school transport injury		
- no injury last year	Reference	
- one or more injuries last year	3.19^a^	2.03; 5.02

Odds ratios with 95 % CI from multiple logistic regression analyses of school cycling injuries adjusted for age, gender, group (i.e. intervention or. control), and baseline level of the potential predictor variables. ^a^collapsed due to few “very unsafe” respondents. Study conducted at three different locations in Denmark, 2010–2011

## Results

General characteristics of the study population averaging 11 years of age at baseline are shown in Table 1 in which it is evident that age and cardiorespiratory fitness were higher in the intervention group (*p* = 0.023 / <0.001, respectively) whereas height (*p* = 0.001) was lower as compared to the control group. The control group was more physically active comparing long term school cycling (*p* = 0.026) and cycling to school (*p* = 0.002) and cycling beyond school (*p* = 0.002) during the last week compared to the intervention group. The pooled 25 %, 50 % and 75 % percentiles for BMI at baseline were 16.3, 17.7 and 19.6.

In Table 2 the results from multivariate adjusted multiple linear and multiple logistic regression analyses of the differences between the intervention and control group in changes in: leisure time physical activity, cycling behaviour cardiorespiratory fitness and weight status, are presented. Except the finding that cardiorespiratory fitness changed in an unfavourable direction in the intervention group compared to the control group (beta: −1.45, *p* < 0.001) no statistically significant different delta values in leisure time physical activity, school cycling or risk of overweight/obesity were identified. Unadjusted changes in leisure time physical activity and cycling behaviour (Additional file 1: Table A) should be interpreted with caution since it was not possible to adjust for the baseline level when using the chi-squared test.

The one year incidence of being involved in a traffic injury was about 25 % and almost half of these injuries occurred during transport to or from school. On average more than 85 % of the total number of injuries happened when cycling and the vast majority of injuries were categorized as solo injuries. On average about 3.5 % of the respondents were involved in a severe injury which involved visiting a hospital (Table 3). No statistically significant differences were observed neither in the incidence of traffic injuries nor in the characteristics of injuries when cross-sectionally comparing the control group and the intervention group in the year preceding this study and in the year during this study (Table 3). When comparing differences in changes in injuries between the control and the intervention group we found no statistical difference in the distribution of proportions of children. The proportion of those who avoided injury at both time points, those who were involved in an injury at either baseline or follow-up and those who were involved in injuries at both time points) differed neither for total injuries (*p* = 0.823) nor for severe injuries (*p* = 0.954).

In Table 4 potential predictors of school transport injuries such as age, child and parental perceived school route safety, travel duration, ethnicity and previous school transport injury were calculated. Except for previous school transport injury (OR: 3.19, *p* < 0.001) none of the investigated covariates were predictors of future school transport injuries.

## Discussion

### Effectiveness

The main finding of this study was that a multifaceted school cycling promotion programme (focusing on structural changes near the school and school cycling motivation) did not affect the degree of school cycling when comparing the differences in the control group with the differences in the intervention group. No effect in weight status was achieved whereas cardiorespiratory fitness changed in an unfavourable direction in the intervention group compared to the control group. It could be speculated that the latter may have been a consequence of more cycling and leisure time physical activity in the control group observed both at baseline (Table 1) and at follow-up (not presented) compared to the intervention group. In order to investigate whether more comprehensive interventions would have contributed to significant changes in transport behaviour we conducted post hoc analysis investigating (through multiple regression analysis adjusted for age, gender and baseline level) if there was a dose response association between cycling to school and the total intensity (including both soft and hard) of interventions. This seemed, however, not to be the case (*p* = 0.356).

### Traffic injuries

Though the incidence of traffic injuries in the present study included both severe and mild injuries the dark figure of injuries (i.e. the gap between injuries that have been registered and injuries that in fact have occurred) was minimized compared to e.g. an approach solely relying on information from emergency departments [21, 22]. We were unable to observe any differences in the frequency of injuries or in the characteristics of the injuries between the control schools and the interventions schools. It should in this connection be noted that we cannot rule out that non-significant differences could be due to the reliability of self-reported traffic injuries or due to implementation issues.

Our data on traffic injuries are unique, independent of the major study challenges (e.g. potential implementation issues) and may be of importance in the future prevention of traffic injuries during transport to school. It is noteworthy that the one year incidence of being involved in a traffic injury was almost 25 % among this age group and that nearly half of these injuries occurred during transport to or from school. On average more than 85 % of the total number of injuries happened during cycling and the vast majority of injuries were categorized as solo injuries. This pattern appear to be stable since observed at both baseline and follow-up and highlights that there is a preventive potential related to school transport injuries. Thus it seems relevant to focus especially on prevention of cycling related injuries. Our data is compatible with a previous study in the sense that the frequency of motor vehicle collision injuries was relatively low among cycling children below 14 years of age [22] in the present study with most incidents categorized as solo incidents or incidents involving a cycling counterpart. Since specific bike lanes has been associated with a lower risk compared to multi-use trails [23] it would, however, in future studies also be relevant to evaluate the potential changes in traffic injuries due to creation of shared trails (used by both pedestrians and cyclists) included in many contemporary urban renewal plans. Predictors of school transport injuries were prospectively identified in analyses of follow-up measures adjusted for the baseline level. Through pooling the intervention and control groups by adjusting for group coherence thereby maximizing statistical strength, we identified that “previous school traffic injuries” was the only statistically significant predictor of future injuries during school transport. For future comparability to other studies it should be reported that 36 of the 54 severe injuries registered at baseline occurred while cycling. A possible preventive strategy could be to teach cycling skills to children who previously have been involved in an injury since it appears that they constitute a high-risk group. Despite the risk of being involved in traffic injuries when actively commuting it should be emphasized that several studies have found the net effect on public health to be positive when valuing the risks from traffic injuries (and air pollution) against the positive health effects [24, 25]. Also it might be speculated if the previous reported “safety in numbers” phenomenon (i.e. when more people cycle then cycling becomes safer) a lowering of injury rates per km [26] is valid. In this study a relatively high school transport injury incidence rate was observed although about two out of three children cycled to school.

### Implementation issues

The findings of this study indicate that the interventions rolled out in this study apparently have been ineffective and that the study may have been subject to a type III error (i.e. a failure to *implement* interventions as planned) and several methodological challenges which appear important to consider in future community based studies are discussed in order to optimize evaluations of future investments in cycling. In several countries large investments are being made in order to increase commuter cycling most likely mainly due to an increased awareness of traffic congestion, emission of pollutants/CO_2_ and physical inactivity. As an example the government in Denmark in 2009 decided to allocate 200 mill $ during a five year period to different projects aimed at improving and increasing cycling [27]. Project “Tryg og Sikker Skolecykling”, was one out of several projects partly funded by this foundation but one of the few projects that were thoroughly evaluated.

The recruitment of public schools into the project was substantially delayed partly due to underestimation of the work required to conduct this milestone as well as replacement of employees. In consequence particularly the schools located in the eastern region/Copenhagen were hasty included into the study before the planned baseline measurements. This clearly had negative effects on the degree of project ownership. It was clear that though the principal of some schools had agreed to participate in the study some teachers involved in the data collection considered the project irrelevant or disruptive. It largely characterises schools with low project ownership that the tasks outsourced to teachers (physical testing and ensuring completion of questionnaires) compromised data quality. We cannot rule out that the large extent of missing values have caused biased results and potentially influenced the external validity. Post hoc complete case analyses (not presented) in which only subjects with full information were included consistently yielded similar results regarding the effectiveness of this intervention. Importantly it should be noted, that hiring more project consultants thus relying less on the willingness of teachers and other stakeholders and keeping a closer contact to the schools contributed to the higher compliance at schools in the western region.

It could be speculated whether actual changes in the degree of school cycling and changes in health parameters could have been blurred in consequence of the low data quality. It is evident from the sample size available in the analyses of changes (requiring information both at baseline and at follow-up) especially were decreased in sample size and thus at a higher risk of being subject to type2-errors. The different distribution of missing values between the schools located in the western vs. eastern part of Denmark reflect some of the challenges experienced in this project (c.f. Additional file 1: Table C).

Originally this study was intended to be carried out as a randomized controlled trial but instead a less rigid but more pragmatic study design had to be used. It was a major challenge to be allowed to control (especially when dictated by the outcome of a randomization) the timing of implementation of expensive structural improvements that had been planned for a long time by the local authorities and sometimes promoted in the local press. We were at first allowed to control the timing of hard cycling interventions aimed at schools in the city of Copenhagen. This unique opportunity was unfortunately missed due to delays in the school recruitment because the timing of baseline measurements became incompatible with the construction work. Furthermore it was evident that we had difficulties passing on the scientific importance that control schools were not offered any cycling interventions. This might have diluted observed effects since some minor interventions were conducted at some control schools (cf. Additional file 1: Table B). Also we acknowledge that relying on a score of the intensity of interventions conducted by The Danish Cyclists Federation is sub-optimal since the estimates might be biased due to the fact that this stakeholder focused primarily on facilitating cycling at the intervention schools.

## Conclusions

This multifaceted school cycling promotion programme did not increase the degree of cycling to school or elicit health beneficial effects among school children. Future school-based intervention studies should attempt to address the implementation issues experienced in this study. No differences were observed neither in the incidence of traffic injuries nor in the characteristics of injuries when comparing the control group and the intervention group. Approximately 50 % of all traffic injuries among this group of children averaging 11 years of age occurred during transport to or from school and most injuries were categorized as solo injuries. Among several investigated potential correlates, previous school transport injury was the only predictor of future school traffic injuries.

## Additional file

Additional file 1:
**Table A.** Unadjusted changes in leisure time physical activity and cycling behaviour. **Table B.** Detailed information on the school based multifaceted interventions. **Table C.** Detailed information on the distribution of available information (i.e. the degree of missing) at baseline questionnaire assessed variables as well as variables assessed through physical testing by the three different regions. (DOCX 22 kb)
