# Supplementary material for: Effectiveness and implementation of interventions to increase commuter cycling to school: a quasi-experimental study
Source: BMC Public Health. 2015 Nov 30;15:1199. doi: 10.1186/s12889-015-2536-1 (PMC4665862; doi:10.1186/s12889-015-2536-1)
Supplement: Additional file 1: — Table A. Unadjusted changes in leisure time physical activity and cycling behaviour. Table B. Detailed information on the school based multifaceted interventions. Table C. Detailed information on the distribution of available information (i.e. the degree of missing) at baseline questionnaire assessed variables as well as variables assessed through physical testing by the three different regions. (DOCX 22 kb) [file 12889_2015_2536_MOESM1_ESM.docx]

# Additional file 1

Table A. Unadjusted changes in leisure time physical activity and cycling behaviour

|  | Control | Intervention | p-value | n  (control / intervention) |
| --- | --- | --- | --- | --- |
|  |  |  |  |  |
| **Change in leisure time  physical activity^*1^** |  |  |  |  |
| -4 (maximal decrease) | 1.5 | 1.7 | 0.689 | 664 / 923 |
| -3 (large decrease | 2.9 | 2.4 |  |  |
| -2 (moderate decrease) | 5.4 | 7.3 |  |  |
| -1 (small decrease) | 11.5 | 12.1 |  |  |
| 0 (unchanged) | 52.7 | 51.8 |  |  |
| +1 (slight increase) | 14.8 | 12.5 |  |  |
| +2 (moderate increase) | 6.8 | 7.5 |  |  |
| +3 (large increase) | 2.1 | 2.8 |  |  |
| +4 (maximal increase) | 2.4 | 2.0 |  |  |
|  |  |  |  |  |
| **Change in long term school cycling ^*2^** |  |  |  |  |
| -2 (maximal decrease) | 3.2 | 3.5 | 0.197 | 663 / 923 |
| -1 (moderate decrease) | 9.5 | 12.4 |  |  |
| 0 (unchanged) | 67.9 | 62.2 |  |  |
| +1 (moderate increase) | 15.1 | 17.0 |  |  |
| +2 (maximal increase) | 4.4 | 5.0 |  |  |
|  |  |  |  |  |
| **Change in cycling last week  beyond school cycling^*3^** |  |  |  |  |
| -3 (maximal decrease) | 1.4 | 1.63 | 0.309 | 663/ 923 |
| -2 (moderate decrease) | 5.1 | 5.2 |  |  |
| -1 (small decrease) | 19.5 | 19.93 |  |  |
| 0 (unchanged) | 43.6 | 40.0 |  |  |
| +1 (small increase) | 24.0 | 23.5 |  |  |
| +2 (moderate increase) | 5.0 | 8.1 |  |  |
| +3 (maximal increase) | 1.5 | 1.6 |  |  |
|  |  |  |  |  |

*Chi-squared tests of differences in distributions in the control group compared to the intervention group for changes in leisure time physical activity, long term school cycling and cycling last week beyond school cycling. All delta variables are defined so that positive values reflect increases and vice versa. Study conducted at three different locations in Denmark, 2010-2011.*

Table B. Detailed information on the school based multifaceted interventions

| **School id** | **School name** | **Intensity of ”soft” interventions** | **Intensity of “hard” interventions** | **Total intervention intensity** |
| --- | --- | --- | --- | --- |
| 1 | Nørrebro Park # | 4/5 | 1 | 5/6 |
| 2 | Valby # | 2 | 2 | 4 |
| 3 | Lergravsparken # | 1/2 | 5 | 6/7 |
| 4 | Kirkebjerg # | 4 | 4 | 8 |
| 5 | Korsager # | 1 | 4 | 5 |
| 6 | Husum | 0 | 0 | 0 |
| 7 | Vibenhus | 0 | 0/1 | 0/1 |
| 8 | Hyltebjerg | 0 | 0 | 0 |
| 9 | Hanssted | 0 | 0 | 0 |
| 10 | Skærbæk #% | 2 | 0 | 2 |
| 11 | Egumvejen #% | 3/4 | 0 | 3/4 |
| 12 | Seden #% | 2/3 | 3 | 5/6 |
| 13 | Skansevejen #% | 3/4 | 3 | 6/7 |
| 14 | Højme #% | 1 | 0 | 1 |
| 15 | Erritsø % | 1 | 0 | 1 |
| 16 | Ebberup % | 0 | 0 | 0 |
| 17 | Nørre Åby #% | 1 | 0 | 1 |
| 18 | Købmagergade % | 0 | 0 | 0 |
| 19 | Hunderup % | 0/1 | 0 | 0/1 |
| 20 | Vestre % | 1 | 0 | 1 |
| 21 | Strib #% | 2 | 0 | 2 |
| 22 | Ørbæk #% | 4 | 0 | 4 |
| 23 | Treldevejens % | 0 | 3 | 3 |
| 24 | Mølkærskolen % | 0 | 0 | 0 |
| 25 | Broskolen % | 0/1 | 0 | 0/1 |

# indicate that school was an intervention school
% indicate that school is located in the western part (Funen and Jutland) of Denmark

The hard interventions affected all children at the school whereas the soft interventions were aimed at the specific school classes. Hard interventions included changes near the school in e.g. road surface, signposting and traffic regulation such as one-way streets and regulation of car commuting drop off zones. Soft interventions included a variety of different school cycling incentives such as school campaigns (n=3), events aimed at the parents (n=3) and/or the children (n=7) also free cycling helmets and cycle gimmicks were provided to some classes. Different scores in the intensity of soft interventions within the same school occurred when the package of soft interventions differed between the participating classes.

Note that the intensity of of interventions was determined as a score from 1-5 by the external collaborator (i.e. The Danish Cyclist Federation) a priori to conduction of the statistical analyses . The higher the score – the more intense the intervention had been.

Table C. Detailed information on the distribution of available information (i.e. the degree of missing) at baseline questionnaire assessed variables as wells as variables assessed through physical testing by the three different regions.

| **Baseline** |  | **East Denmark** | **West Denmark** |
| --- | --- | --- | --- |
| Questionnaire |  | 65.6 | 91.2 |
| Physical testing |  | 72.8 | 89.3 |
|  |  |  |  |
| **Follow-up** |  |  |  |
| Questionnaire |  | 60.8 | 80.3 |
| Physical testing |  | 66.0 | 76.0 |

All numbers are reported as percentages calculated relative to the total sample of children from West (n=1401) and East (n=1000) included into the study.

Schools in city of Copenhagen are defined as eastern region whereas schools in the island of Funen and the City of Fredericia are defined as western region.

Percentages reflecting the degree of missing information in questionnaire assessed variables are based on the question regarding long term school cycling (which at baseline was equivalent to the response rate in the other questions, cf. Table 1). Percentages reflecting the degree of missing information in physical tests are based on measurements of height (which at baseline was equivalent to the rate in the other physical tests, cf. Table 1).
